# Seroprevalence of *Pasteurella multocida* Serotype A2 in Cattle, Afar Region, Ethiopia

**DOI:** 10.1155/vmi/6610210

**Published:** 2025-05-09

**Authors:** Fanuel Bizuayehu Yihunie, Ashenafi Syoum, Teshager Dubie

**Affiliations:** Department of Veterinary Medicine, College of Veterinary Medicine and Animal Science, Samara University, Semara, Ethiopia

**Keywords:** Afar, cattle, *Pasteurella multocida*, seroprevalence

## Abstract

Bovine respiratory disease (BRD), also known as shipping fever, is an economically significant disease affecting cattle in Ethiopia and is caused by the *Pasteurella* species. There is limited information on the serological prevalence of these species in Ethiopia. This study aimed to identify serotype A2 and estimate the seroprevalence of *Pasteurella multocida* in cattle in the Asayita, Dubti, and Chifra districts of Afar Region, Northeastern Ethiopia. A cross-sectional study was conducted on randomly selected cattle from November 2023 to June 2024. Blood samples were collected from nonvaccinated bovine species, recording sex, age, and body condition. Descriptive statistical analysis was used to summarize the results, with data managed using Microsoft Excel and analyzed with SPSS Version 20. Descriptive statistics were employed to determine the occurrence of the serotype. The chi-square test was employed to determine the association between variables. 93.8% of tested blood samples were found positive for *Pasteurella multocida* serotype A2. Among the factors tested, body condition scores showed a significant association (*x*^2^ (2, *N* = 384) = 34.07, *p* ≤ 0.001) with the prevalence of the serotype. This study provides valuable insights into the seroprevalence and serotype distribution of *Pasteurella multocida* in the study area, highlighting the need for targeted control measures.

## 1. Introduction

Pasteurellosis in cattle, also known as shipping or transit fever, is a serious and highly contagious bacterial infection. It primarily affects the respiratory system, causing symptoms such as pneumonia, fever, and septicemia [1]. The disease is caused by two main bacteria: *Mannheimia (Pasteurella) haemolytica* and *Pasteurella multocida*. *P. multocida* is a common pathogen, often found in the nasal passages of healthy carrier animals. Stress factors such as transportation, overcrowding, and mixing with new animals can trigger outbreaks [2]. *P. multocida* is usually a secondary pathogen that invades pre-existing damaged airways.

*Pasteurella multocida* is the most commonly isolated bacterial pathogen from the lungs of cattle. Normally, *P. multocida* is confined to the respiratory tract residing in the nasopharyngeal area of cattle [1, 3]. However, following stress or viral infection, *P. multocida*'s replication rate in the upper respiratory tract can increase significantly, enhancing the likelihood of culturing the bacteria. This increase in bacterial growth, followed by inhalation and colonization of the lungs, may be due to the suppression of the host's defense mechanisms by environmental stressors or other infections [3].

*Pasteurella multocida* strains are categorized into five capsular serogroups (A, B, D, E, and F), based on Carter's 1955 indirect hemagglutination test. Additionally, these strains are divided into 16 somatic or lipopolysaccharide (LPS) serotypes using Heddleston's gel diffusion precipitation assay [4]. Among these, the most prevalent and pathogenic serotypes of *P. multocida* are A1, A3, and A4 [5]. These serovars are frequently isolated from the nasopharynx of healthy cattle. Serovar A2 is a common pathogen in sheep and has traditionally been considered a commensal organism in cattle. However, evidence suggests that serovar A2 may not be entirely commensal in cattle, indicating a potentially more active role in disease than previously thought [6, 7].

The status of seroprevalence of *P. multocida* and pasteurellosis in Ethiopia is limited. A study in selected areas of Ethiopia indicated a prevalence rate of 15.25% [5]. Research on the distribution of the serotypes within Ethiopia, particularly in the Afar Region, is scarce. According to the district veterinarian in the Afar Region, ruminant pasteurellosis remains a significant health challenge. However, there is no definitive evidence of the seroprevalence, serotypes, or strains responsible for pasteurellosis in cattle in the region. Ongoing efforts focus on incorporating various field strains into vaccine development in Ethiopia. In light of this, the current study seeks to identify the serotype A2, estimate seroprevalence, and explore potential risk factors associated with *P. multocida* serotype A2 in cattle from selected districts in the Afar Region.

## 2. Materials and Methods

### 2.1. Description of the Study Area

The research was carried out from November 2023 to June 2024 in the zone one districts of the Afar Region, namely, Asayita, Dubti, and Chifra. The Afar pastoral region lies between 39°34′ and 42°28′ East longitude and 8°49′ and 14°30′ North latitude. The region covers roughly 270,000 km^2^. It borders Eritrea to the northeast and Djibouti to the east, and it also meets regional borders with Tigray, Amhara, Oromia, and Somali. The climate ranges from arid to semiarid, with sparse and unpredictable rainfall. The region experiences bimodal rainfall patterns, with an average annual rainfall of less than 500 mm on the semiarid western escarpments, which decreases to 150 mm in the more arid eastern areas. Altitudes vary from 120 m below sea level in the Danakil Depression to 1500 m above sea level. Temperature ranges span from 20°C at higher altitudes to 48°C in lower areas. The Afar Region has a population of 1.8 million, predominantly pastoralists, according to the Central Statistical Agency (CSA) in 2021 [8].

### 2.2. Study Design and Population

A cross-sectional study was conducted from November 2023 to June 2024. A simple random sampling method was employed to include the study animals. The study included all cattle regardless of breed, sex, age group, body condition, and management systems. All information from the sampled animal was recorded. All cattle except those that were vaccinated and below 6 months of age were included in the study.

### 2.3. Sampling and Sample Size Determination

Simple random sampling was employed to collect blood samples from individual animals. Animals that had been vaccinated were excluded from the study. Sampling was distributed proportionally based on the total population in the study district's kebeles. To determine the sample size necessary for estimating seroprevalence, we used the formula provided by Thrusfield (2005). We considered a prevalence rate of 50%, an absolute precision of 5%, and a confidence level of 95%. Consequently, a total of 384 cattle were sampled.

(1) $$\begin{array}{c} n=\frac{1.96^{2}\left(P_{\exp}\right)\left(1-P_{\exp}\right)}{d^{2}},\\ n=\frac{1.96^{2}\left(0.5\right)\left(1-0.5\right)}{0.05^{2}},\\ n=384, \end{array}$$

where *n* = sample size, 1.96 = the value of “*Z*” at a 95% confidence interval, *P*_exp_ = expected prevalence, and *d* = desired absolute precision.

### 2.4. Sample Collection and Transportation

Blood samples of approximately 5 to 10 mL were collected from each animal through jugular vein puncture using plain vacutainer tubes. This was done after properly restraining the animals and disinfecting the site with 70% alcohol. Additionally, information such as the animal's sex, age, breed, body condition, antibiotic treatment, and vaccination history was recorded. Each sample was labeled with specific standard identification and transported to Samara University Veterinary Microbiology Laboratory for serum collection. The tubes were set tilted overnight at room temperature to allow clotting, and the next morning, sera were harvested from the clotted blood using sterile and labeled cryovial tubes.

### 2.5. Serological Analysis

Antibodies against *P*. *multocida* serogroup A2 were measured using a specific antigen-containing kit using the indirect hemagglutination test (IHA) [9]. Briefly, two-fold dilutions of the test sera starting from 1:5 to 1:640 were made in normal saline using microtiter plates (96 wells), and the 25 μL amount was added to all the wells of the plate except those of columns 11 and 12, which served as control. The first four wells (A–D) of column 11 were added with known negative serum and the last four wells (E–H) with the known positive serum. Sensitized RBCs (1%) were added in equal amounts (25 μL) to all the wells of the plate so that column 12 served as a control for the RBCs. Then, the plates were incubated at room temperature for 2 hours, and the observations were recorded.

### 2.6. Data Management and Analysis

Data obtained from the investigations were coded and entered into Microsoft Excel and analyzed using SPSS Version 20 software. Descriptive statistics were used to summarize the seroprevalence of *P. multocida* A2 isolate across different factors. The chi-square test of independence was used to determine any association between the seroprevalence and factors. Variables with *p* value < 0.05 were considered statistically significant.

### 2.7. Ethical Approval

This study was approved by the ethics review committee of Samara University (ERC-57/2023). The studies were conducted in accordance with the local legislation and institutional requirements.

## 3. Result and Discussion

In the present study, 274 (71.4%) female and 110 (28.6%) male animals were included. From this, 201 (52.3%) were adult and 183 (47.7%) were young. The body condition score of the study animals comprises 99 (25.8%), 161 (41.9), and 124 (32.3%) good, moderate, and poor animals, respectively. In this study, out of 384 serum samples examined, 360 (93.8%) were seropositive for *P. multocida* serotype A2 (Table 1).

The study indicated no significant association between the seroprevalence of *P. multocida* serotype A2 and sex, age, and study districts. However, there is a significant relationship between body condition score and the seroprevalence of pasteurellosis, *x*^2^ (2, *N* = 384) = 34.07, *p* ≤ 0.001. Cattle with poor body condition were more likely to be seropositive than medium and good body condition cattle (100% to 96.3% and 81.8%).

Regardless of the age group, sex, and district difference, the current study presents a 93.8% seroprevalence of *P. multocida* A2 in selected districts of the Afar Region. The finding is indicative of previous infection of the herds. The high prevalence in this study period may be associated with stress due to the scarcity of food in the region, which can aggravate the infection level in the herd. Additionally, the high seropositivity observed may be due to factors such as fewer grazing lands, which may favor outbreaks. The finding is unlikely compared to previous studies conducted in selected regions of Ethiopia [5] and Assam, India [10]. The variation may arise due to regional differences and the type of production in animals. Vaccination may also contribute to the difference in seropositivity [11]. Since animal production is essential for the rural population in Afar, vaccination against pasteurellosis is an important way to reduce losses due to production and death of the animal.

Although serotype B:2 has been mainly reported in Asian countries and E:2 in African countries, both serotypes have recovered in African countries [12]. However, the identified serotype in this study was serotype A2. However, it is very important to cover a wide epidemiological study area to conclude these findings.

## 4. Conclusion

A high prevalence of *P. multocida* serotype A:2 was recorded in the study areas. The seroprevalence correlated with the animals' body condition, showing a higher prevalence in those with poor body condition. Consequently, the current findings recommend further extensive studies on *P. multocida* serotypes across broader districts of the region. This would aid in understanding its impact and assessing the serotype diversity of *Pasteurella* infections in cattle to formulate an effective control strategy.

## Figures and Tables

**Table 1:** Seroprevalence of *P. multocida* serotype A2 and its association with different factors.

Factors		Total examined	Positive	*X* ^2^	*p* value
Sex	Female	274 (71.4%)	259 (94.5%)	0.982	0.32
	Male	110 (28.6%)	101 (91.8%)		

Age	Young	183 (47.7%)	174 (95.1%)	1.059	0.3
	Adult	201 (52.3%)	186 (92.5%)		

Body condition	Poor	124 (32.3%)	124 (100%)	34.07	≤ 0.001
	Moderate	161 (41.9%)	155 (96.3%)		
	Good	99 (25.8%)	81 (81.8%)		

District	Asayita	145 (37.8%)	136 (93.8%)	0.08	0.99
	Dubti	130 (33.9%)	122 (93.8%)		
	Chifra	109 (28.4%)	102 (93.6%)		

Total		384 (100%)	360 (93.8%)		

## Data Availability

The data that support the findings of this study are available from the corresponding author upon reasonable request.
